# Technical note: Identification system for production line tracking of pharmaceuticals

**DOI:** 10.1155/S1463924693000112

**Published:** 1993


### Technical note: Identification system for production line tracking of pharmaceuticals

Radio frequency identification (RFID) is a relatively new technology which is now finding applications in the pharmaceuticals industry. Texas Instruments has set up a separate operation called TIRIS (for Texas Instruments Radio Identification Systems) to promote its RFID system. The TIRIS RFID system is a battery-less low-frequency technology that electronically recognizes, and tracks passive transponder tags. It operates over short distances without line of sight or direct contact. TIRIS tags can store 20 digits or 64 bits or more of data and are available in both read-only and read/write (i.e. updatable) versions.

TIRIS has been working with Pierrel-Ospedali, an Italian pharmaceutical company, which was looking for ways to improve the production process for its intravenous solutions for which it required the highest standards of quality and sterilization. The result was an innovative and reliable system using small RFID ID tags embedded in Teflon blocks attached to the top of each stainless-steel sterilization rack.

The plant houses a production line that bottles medical solutions, for example glucose. The bottles move through production on large stainless-steel racks measuring $1{\cdot}2 \times 1{\cdot}2 \times 1{\cdot}3$ m high and each holding 1000 bottles. The racks are tracked in batches of five as they enter an autoclave oven for sterilization at 120 °C or above. If there is any doubt as to the quality of the sterilization process, then the batch of bottles is destroyed.

Prior to the automation of this line, most product tracking and quality-control measures were performed manually. Employees pushed the racks from one location to another, into the autoclave, and then to an unload point. This clearly led to some operator errors.

A conveyor system was installed to automatically move the racks. Bar-coding the racks was considered, but rejected due to the harsh conditions of the sterilization process. Radio frequency identification was chosen because:

(1) The RFID tags could withstand the high temperatures of the autoclave and did not require contact, nor line-of-sight to be read.

(2) RFID offered the possibility of upgrading.

Small transponders were inserted into Teflon blocks attached to the top corner of each stainless-steel rack. Readers were positioned along the conveyor system to 'interrogate' the tags and capture the unique ID number of each rack as it passed. With read/write tags, the need to store all information in a central computer is reduced: the tags themselves can act as data carriers.

*For further information about the TIRIS RFID system or the Pierrel application contact David Hyslop, Texas Instruments, Manton Lane, Bedford, Bedfordshire MK41 7PA, UK.*

